# *FocSge1* in *Fusarium oxysporum* f. sp. *cubense* race 1 is essential for full virulence

**DOI:** 10.1186/s12866-020-01936-y

**Published:** 2020-08-14

**Authors:** Vartika Gurdaswani, Siddhesh B. Ghag, Thumballi R. Ganapathi

**Affiliations:** 1 School of Biological Sciences, UM-DAE Centre for Excellence in Basic Sciences, University of Mumbai campus, Kalina, Santacruz (E), Mumbai, 400 098 India; 2 Plant Cell Culture Technology Section, Nuclear Agriculture & Biotechnology Division, Bhabha Atomic Research Centre, Trombay, Mumbai, 400 085 India

**Keywords:** *Fusarium oxysporum* f. sp. *cubense*, Banana, Fusarium wilt, Virulence, *FocSge1*, Transcription factor, SIX1

## Abstract

**Background:**

Fusarium wilt disease of banana is one of the most devastating diseases and was responsible for destroying banana plantations in the late nineteenth century. *Fusarium oxysporum* f. sp. *cubense* is the causative agent. Presently, both race 1 and 4 strains of Foc are creating havoc in the major banana-growing regions of the world. There is an urgent need to devise strategies to control this disease; that is possible only after a thorough understanding of the molecular basis of this disease.

**Results:**

There are a few regulators of Foc pathogenicity which are triggered during this infection, among which Sge1 (Six Gene Expression 1) regulates the expression of effector genes. The protein sequence is conserved in both race 1 and 4 strains of Foc indicating that this gene is vital for pathogenesis. The deletion mutant, *FocSge1* displayed poor conidial count, loss of hydrophobicity, reduced pigmentation, decrease in fusaric acid production and pathogenicity as compared to the wild-type and genetically complemented strain. Furthermore, the C-terminal domain of FocSge1 protein is crucial for its activity as deletion of this region results in a knockout-like phenotype.

**Conclusion:**

These results indicated that *FocSge1* plays a critical role in normal growth and pathogenicity with the C-terminal domain being crucial for its activity.

## Background

Banana (*Musa* spp.) is one of the important staple food crops of the world. However, the production is constrained largely due to diseases including the Fusarium wilt disease caused by *Fusarium oxysporum* f. sp. *cubense* (Foc). Physiologically, Foc is divided into four races, of which race 1 and Tropical race 4 (TR4) are important. Foc race 1 caused havoc in the late nineteenth century resulting in the shifting of the banana industry from race 1-susceptible Gros Michel variety to the race 1-resistant Cavendish variety [1]. However, the recently evolved TR4 strain infects almost all edible cultivars of banana including the race 1-resistant Cavendish; and thus far, no known substitute for Cavendish exists. And, currently no known effective strategies to curb this pathogen have been offered from any quarters (industry or research).

In the absence of its host (banana plant) Foc remains in the soil as a saprophyte or survives as dormant chlamydospores [2]. In the presence of banana roots, Foc switches to the pathogenic mode and colonizes it. During this switch, there is a complete reprogramming of the expression of genes required for pathogenicity such as cell wall-degrading enzymes, effector proteins and toxic secondary metabolites. However, this transition phase is governed by a few master transcriptional regulators which further control the gene expression profile. A similar morphological switching was found to be controlled by *WOR1* from a white to opaque cell type in *Candida albicans* and *RYP1* from filamentous to a yeast form in *Histoplasma capsulatum*. *Wor1* orthologs have been identified in phytopathogenic fungi that play a crucial role in pathogenicity. *Fusarium graminearum Fgp1* and *Botrytis cinerea Reg1* genes regulate pathogenicity and trichothecene toxin synthesis [3, 4]. *Sge1*, a homolog of *Wor1* was first identified in *Fusarium oxysporum* f. sp. *lycopercisi* (Fol) and was shown to be required for parasitic growth and virulence in tomato [5]. Deletion or inactivation of SGE1 resulted in reduced pathogenicity and was further correlated with the reduced levels of toxin production and effector proteins.

SGE1 from Foc TR4 was shown to regulate a series of genes required for infection. An SGE1 deletion mutant of Foc TR4 demonstrated impaired conidiation and complete loss of virulence on banana plantlets [6]. However, *Sge1* deletion mutant of Fol showed no effect on colonization capability, whereas colonization was compromised in *Sge1* mutant of Foc. A homolog of *Sge1* in *Fusarium verticillioides* (SGE1) is required for pathogenicity and synthesis of secondary metabolites such as fumonisins and fusarins [7]. Nonetheless, SGE1 in *F. verticillioides* was not essential for conidiation as it was in Foc and Fol. This indicates that SGE1 has multiple yet different roles in different phytopathogenic fungi. *Sge1* is known to regulate the expression of major effector genes called *SIX* (secreted in xylem) which are synthesized and secreted into the xylem of the infected tomato plant [5]. Among the SIX proteins, SIX1 is most crucial as its deletion led to reduced virulence of Foc TR4 on banana plants [8], *F*. *oxysporum* f. sp. *conglutinans* on cabbage [9] and *F*. *oxysporum* f. sp. *lycopersici* on tomato [10]. Furthermore, even though SIX proteins are homologous, they are host-selective and inefficient to complement in other pathotypes [9]. This clearly indicates that the interaction of the host plant with the pathogenic strain is differential and intricate. Thus, deciphering the virulence in each pathosystem is critical for a thorough understanding of the infection paradigm.

Pathogen infection strategies and molecular pathways in pathogenesis have always been a subject of rigorous exploration and research. Newer perceptions in this direction will further help in developing appropriate disease management strategies. Depending on the previously described variable, but critical roles of *Sge1* homologs in pathogenicity and development, we sought to investigate the role of *FocSge1* in Foc race 1. Our results demonstrate that *FocSge1* is required for conidiation, pigmentation, colony hydrophobicity and full virulence on banana plants.

## Results

### Identification of *FocSge1* in *F*. *oxysporum* f. sp. *cubense*

**Fig. 1:**
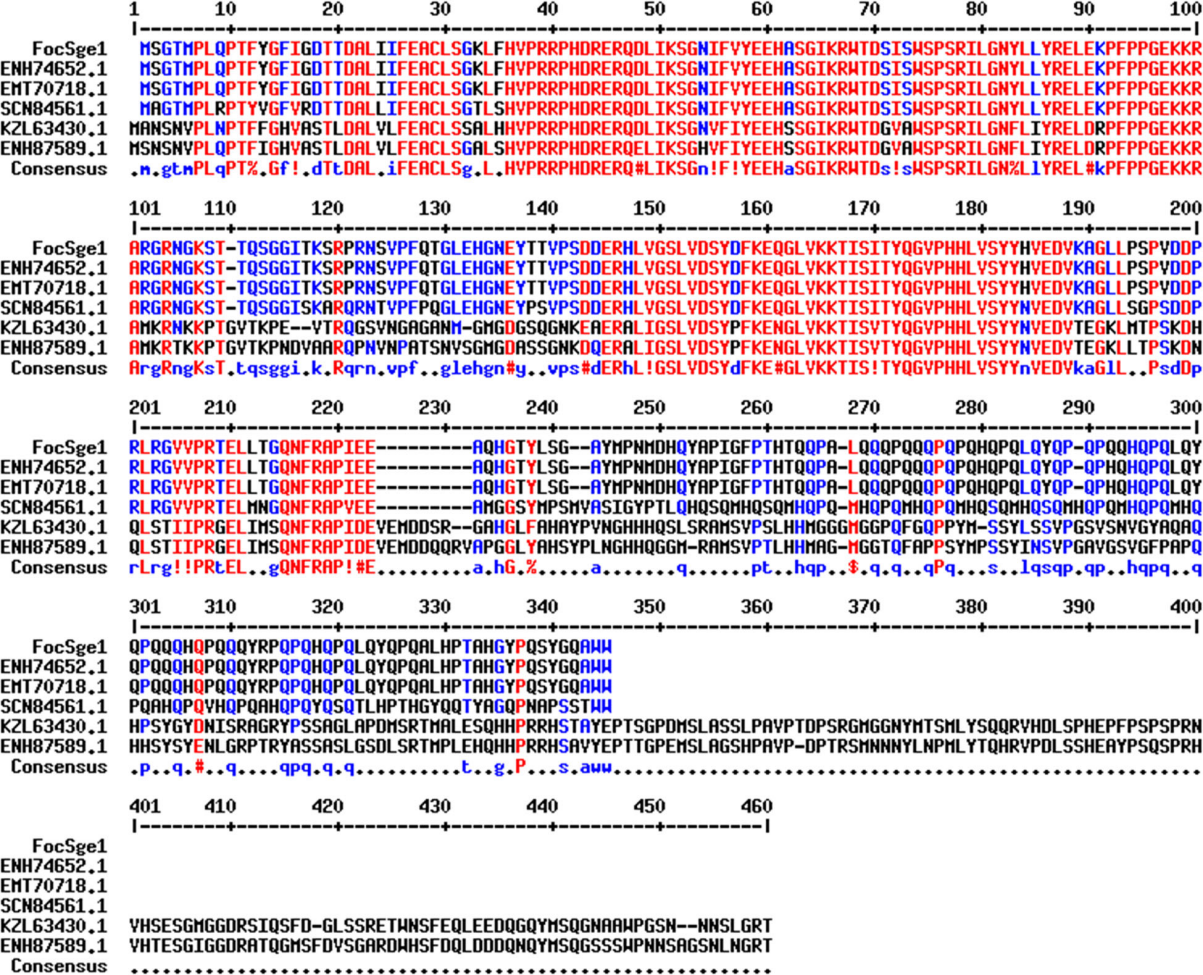
Multiple sequence alignment of FocSge1 protein with hypothetical protein having Gti-Pac2 domain from Foc race 1 (ENH74652), hypothetical protein with Gti-Pac2 domain from Foc race 4 (EMT70718), related to *S*. *pombe* pac2 protein from *Fusarium fujikuroi* (SCN84561), Gti1/Pac2 family protein from *Colletotrichum tofieldiae* (KZL63430) and camp independent regulatory protein from *Colletotrichum orbiculare* (ENH87589)

*FocSge1* gene was PCR amplified from the genomic DNA of Foc race 1 and sequenced. The coding sequence of *FocSge1* was translated in silico to its corresponding amino acid sequence using ExPasy translate tool and used for multiple sequence alignment with its closest homologs. FocSge1 is a 330 aa protein with a theoretical pI of 8.47. It has a Gti-Pac2 domain at the N-terminal end. The Gti1 protein in *Schizosaccharomyces pombe* helps in utilization of gluconate under glucose starvation condition whereas Pac2 protein controls the sexual development in a pathway independent of the cAMP cascade. A multiple sequence alignment of FocSge1 with hypothetical proteins containing Gti-Pac2 domain from Foc race 1 (ENH74652), race 4 (EMT70718), related to *S*. *pombe* pac2 protein from *Fusarium fujikuroi* (SCN84561), Gti1/Pac2 family protein from *Colletotrichum tofieldiae* (KZL63430) and cAMP-independent regulatory protein from *Colletotrichum orbiculare* (ENH87589) showed a high level similarity (Fig. 1). Gti/Pac2 domain is also shared by some crucial transcription factors involved in dimorphic switching. A multiple sequence alignment of FocSge1 protein and gluconate transporter inducer Gti1 from *S*. *pombe* (NP_592911) was performed with Bcreg1 from *Botrytis cinerea* (XP_024547925), Ryp1 from *Histoplasma capsulatum* (ABX74945.1) and Wor1p from *Candida albicans* SC5314 (AOW26645.1) (Supplementary Figure S1). These sequences were found to be conserved at the N-terminal end (1–120 amino acid residues). The two motifs KRWTD and WSPSR were highly conserved in all the five sequences analyzed. This indicates that FocSge1 is an important protein involved in switching of lifestyles, probably from saprophytic to parasitic mode in Foc race1. Being crucial, the protein could be conserved in most of the *Fusarium oxysporum* strains. Hence, a multiple sequence alignment of protein sequences from *Fusarium oxysporum* f. sp. *lycopersici* 4287 (XP_018248224), *Fusarium* sp. FOSC 3-a (EWY91661), *Fusarium oxysporum* f. sp. *pisi* HDV247 (EXA45922), *Fusarium oxysporum* Fo5176 (EGU84598) and *Fusarium oxysporum* Fo47 (EWZ38328) was performed. The Sge1 homologs in these strains are identical except for 6 mismatches among them and an additional proline-glutamine residues in Fo47 strain at 306–307 position in the primary sequence (Supplementary Figure S2).

**Fig. 2:**
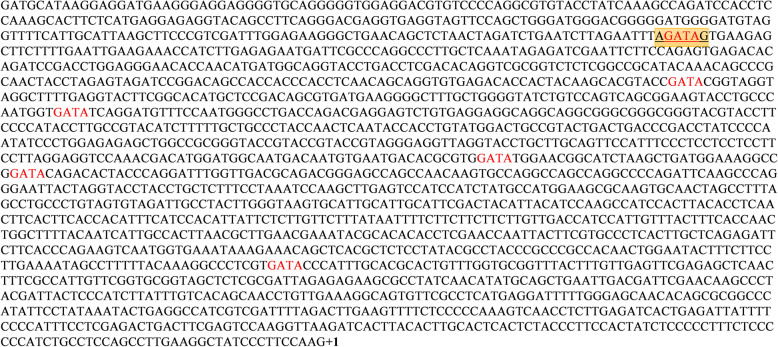
Promoter analysis of *FocSge1* gene showing single GATA transcription factor recognition site

The 2 kbp upstream sequence of *FocSge1* was analyzed for the presence of GATA element. GATA factors bind to a conserved signature element WGATAR (W = T or A and R = G or A) present in the regulatory region of the gene. In *FocSge1* upstream sequence we found one GATA element with sequence AGATAG (Fig. 2). GATA factors are regulated by the type of nitrogen source in the medium which in turn control the expression of genes that are present downstream of this element. Since GATA element was identified in *FocSge1* upstream region, it is likely that the expression of *FocSge1* is indirectly regulated by the nitrogen sources.

### Targeted deletion of *FocSge1* gene and complementation

**Fig. 3:**
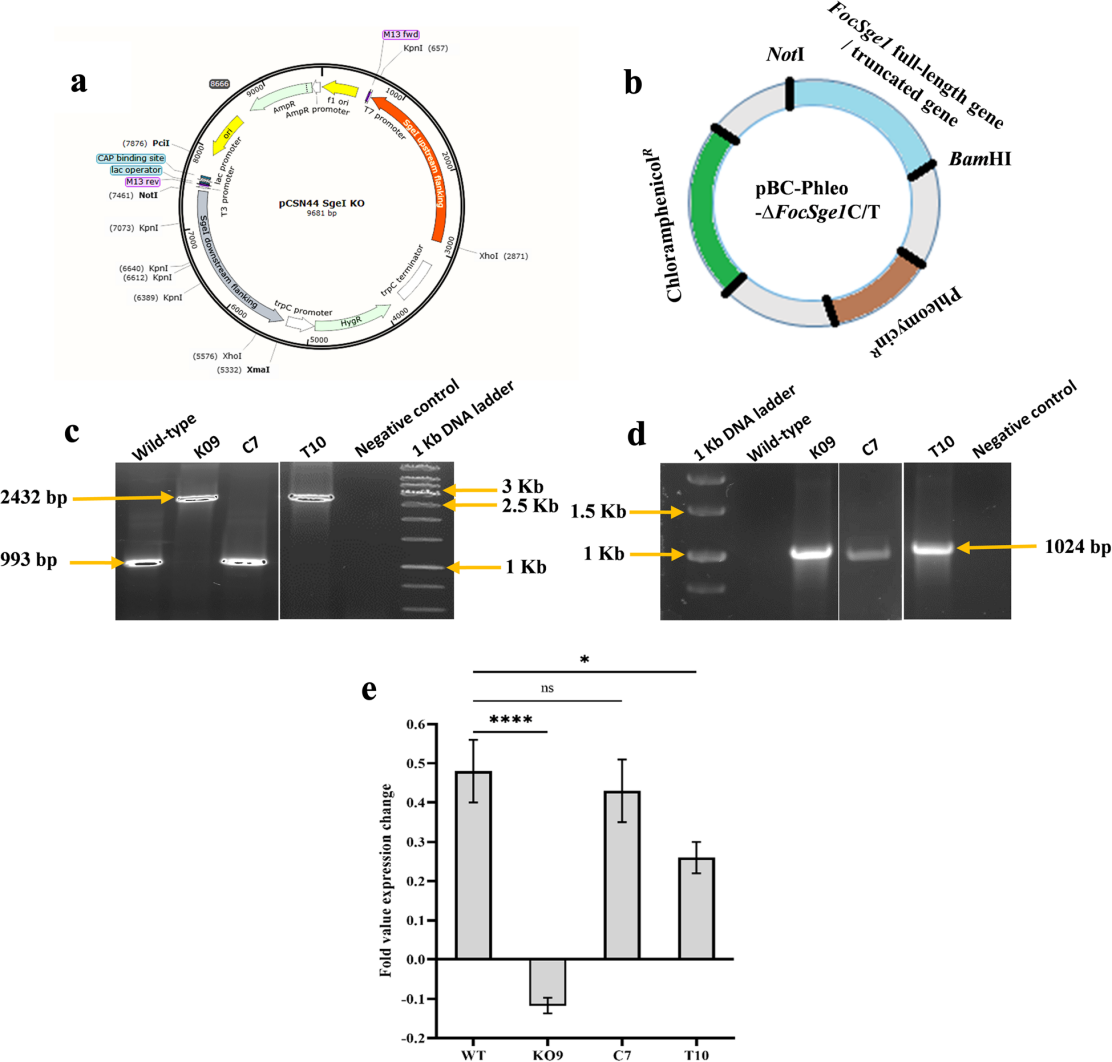
Construction of pCSN44-∆*FocSge1* deletion vector (**a**) and pBC-Phleo ∆*FocSge1C/T* complementation or truncated vector (**b**). Screening of the transformants using *FocSge1* gene coding sequence primers (**c**) and hygromycin resistant gene coding sequence primers (**d**). Quantitative real-time RT-PCR showed differential expression of *FocSge1* gene in wild-type, complementation mutant C7 strain and truncated mutant T10 strain in presence of banana cells whereas no expression was detected in deletion mutant exposed to banana cells (**e**). Dunnett’s multiple comparisons test was performed to determine the significance between the expression levels of wild-type and mutant strains (**** *P* < 0.0001, * *P* < 0.05, ns- non-significant)

To investigate the function of *FocSge1*, pCSN44-∆*FocSge1* deletion vector was transformed into Foc race 1 spheroplasts and transformants obtained through homologous recombination were selected on hygromycin-containing medium (Fig. 3a). First fifteen transformants obtained were screened for the absence of *FocSge1* coding sequence by PCR using *FocSge1*cds Fw / *FocSge1*cds Rv primer set (Fig. 3c). Two transformants K09 and K12 were confirmed knockout *FocSge1* transformants. These transformants were further confirmed for the presence of the hygromycin coding sequence for an amplicons of 1024 bp using PCR (Fig. 3d). Among the two transformants, K09 was selected for further analysis. The K09 deletion mutant was complemented with the full-length *FocSge1* gene or its truncated version and the transformants were selected on phleomycin. Of the several transformants obtained, one full-length (C7) and one truncated mutant (T10) were selected for further analysis. The complemented mutant strains were screened for the presence of the *FocSge1* coding sequence by PCR. An amplified fragment of 993 bp in full-length complementation strain comparable with the wild-type and 780 bp in truncated mutant was observed that was completely absent in the K09 deletion mutant strain (Fig. 3c).

### Relative expression analysis of *FocSge1*

To check the expression of *FocSge1* gene in the wild-type and mutant strains, real-time PCR (RT-PCR) was carried out. Cell suspension cultures of banana cv. Rasthali inoculated with conidia were assessed after 60 h for the expression. The expression of *FocSge1* was induced in wild-type strain infected banana cells whereas no expression was seen in the deletion stain K09. However, the expression was restored in the complementation and truncated strain, C7 and T10 respectively (Fig. 3e).

### Effects of *FocSge1* on hyphal growth, conidiation, hydrophobicity and pigmentation

**Fig. 4:**
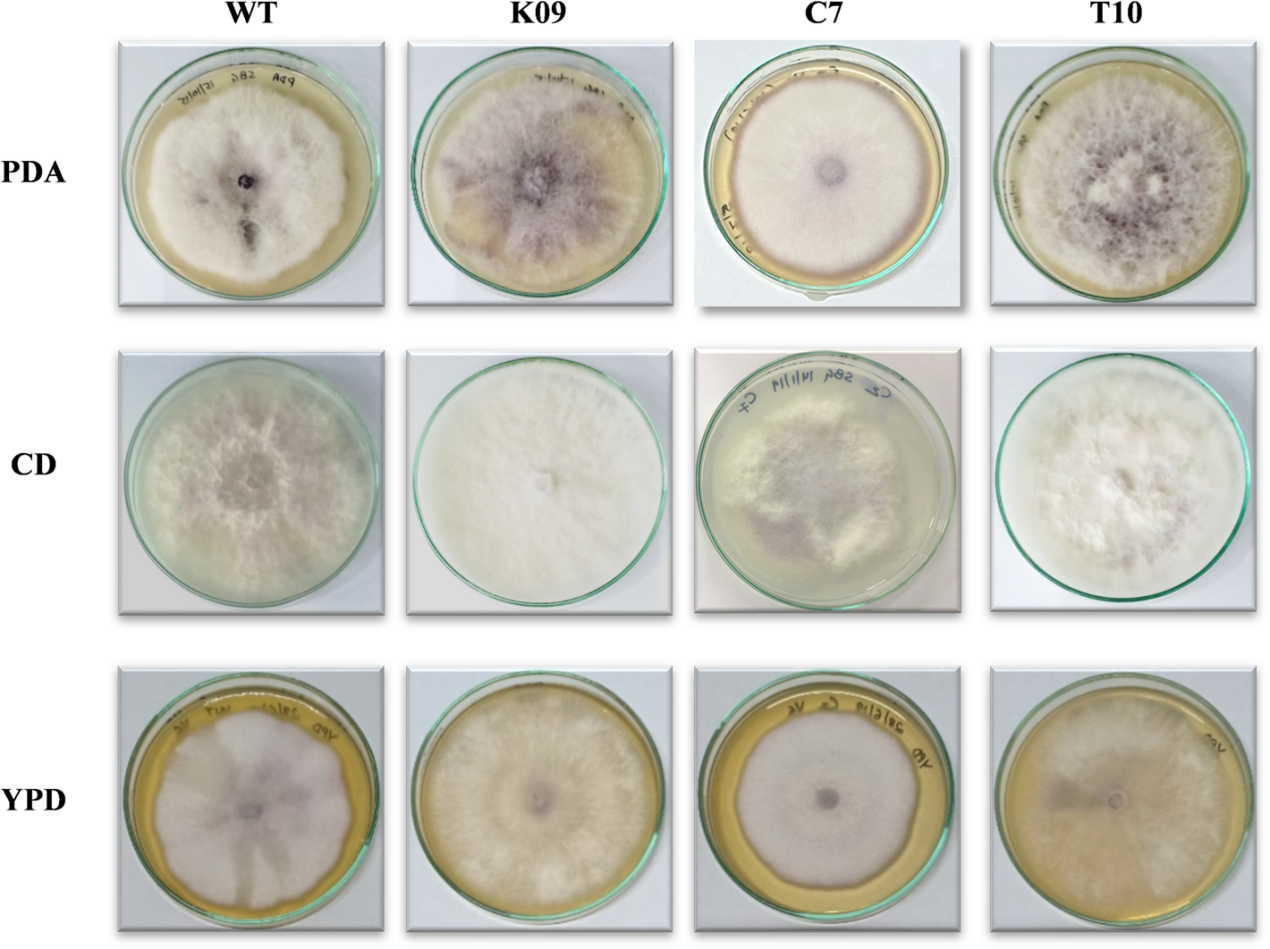
Growth of wild-type and mutants K09, C7 and T10 on different medium such as PDA, Czapek Dox and YPD

**Fig. 5:**
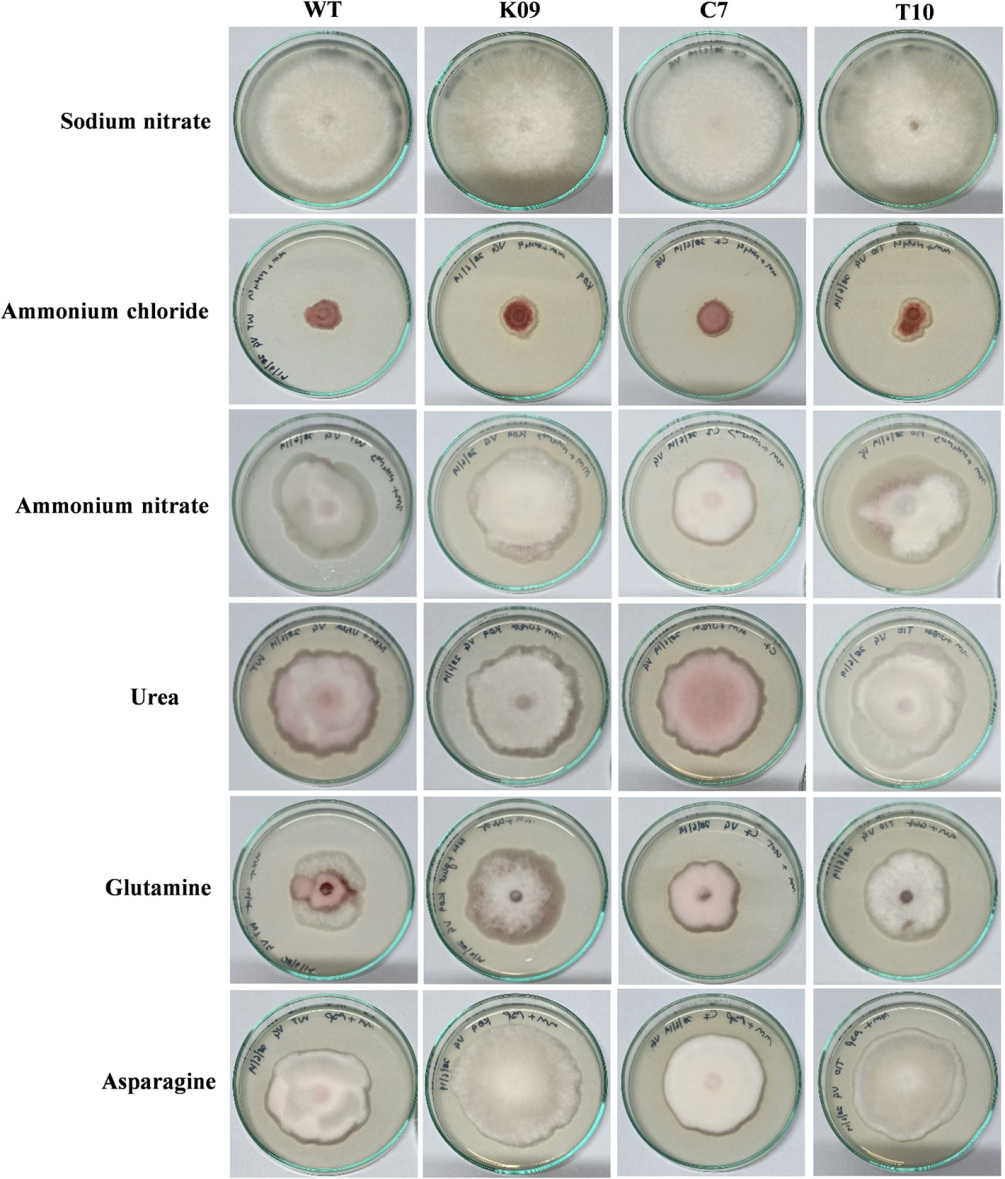
Growth morphology of the wild-type strain and mutants K09, C7 and T10 on minimal medium supplemented with different nitrogen sources

The deletion of *FocSge1* didn’t affect the radial growth of any of the transformants as compared to the wild-type strain. However, it dramatically affected conidial count, colony hydrophobicity and pigmentation. The K09 deletion mutant strain showed reduced aerial mycelium on potato dextrose agar (PDA) medium as compared to the wild-type strain and the full-length complemented strain C7. The truncated mutant T10 showed growth phenotype similar to that of the deletion mutant. Further, the growth characteristics of the wild-type and C7 strains were comparable on PDA, Czapek-Dox and YPD agar media, whereas the K09 and T10 behaved alike (Fig. 4). Since we assume that *FocSge1* gene is regulated by nitrogen sources, the effect of different nitrogen sources on Foc growth was then assessed. Different nitrogen sources such as sodium nitrate, ammonium chloride, ammonium nitrate, urea and amino acids such as glutamine and asparagine were used for growth analysis. The kind of growth and pigmentation obtained in wild-type strain was very different from that of the deletion mutant K09 and truncated mutant T10 in all the media. As expected, the phenotype was reverted in the genetic complementation mutant C7. (Fig. 5).

**Fig. 6:**
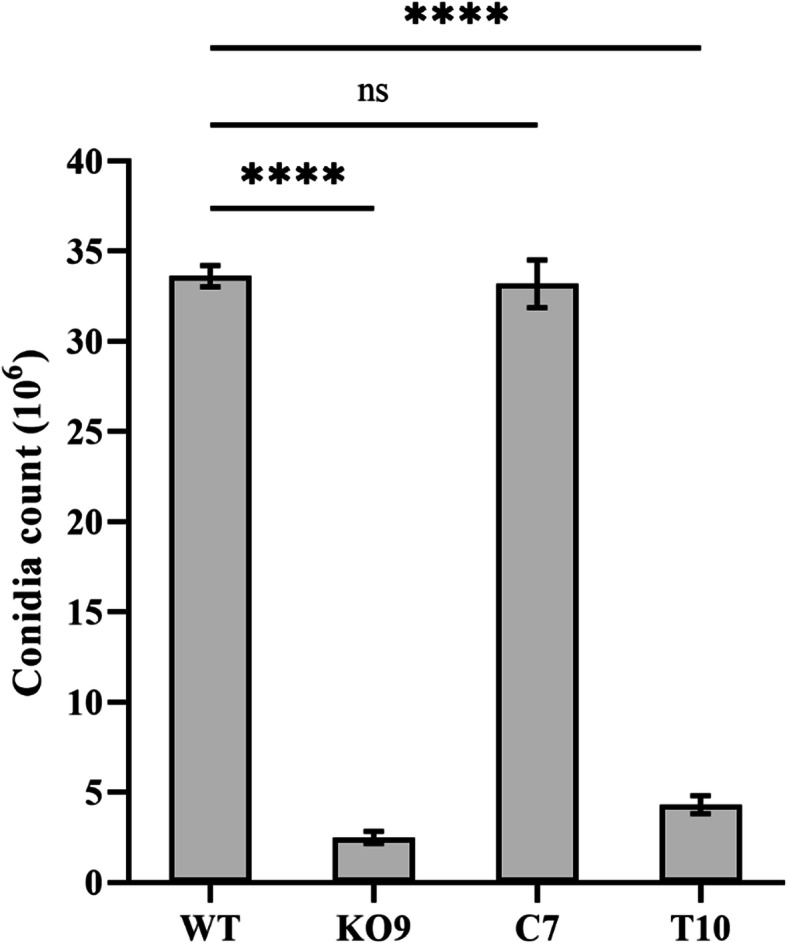
Conidial count of the wild-type strain and mutants K09, C7 and T10 where deletion mutant and truncated mutant demonstrated reduced conidial count. Dunnett’s multiple comparisons test was performed to determine the significance between the conidial count of wild-type and mutant strains (**** *P* < 0.0001, ns- non-significant)

The number of conidia produced by the deletion mutant K09 (2.5 × 10^6^) was significantly reduced when compared with the wild-type strain (3.3 × 10^7^). However, this reduced conidial count was completely restored upon genetic complementation with the full-length *FocSge1* gene as evident in the C7 mutant. The truncated mutant T10 also exhibited low conidial count comparable with the deletion mutant (Fig. 6). Nevertheless, there was no phenotypic abnormality seen in the appearance of the conidia derived from all the three mutants. Furthermore, the conidia produced by all the three mutants were viable and capable of germinating with equal efficiency.

**Fig. 7:**
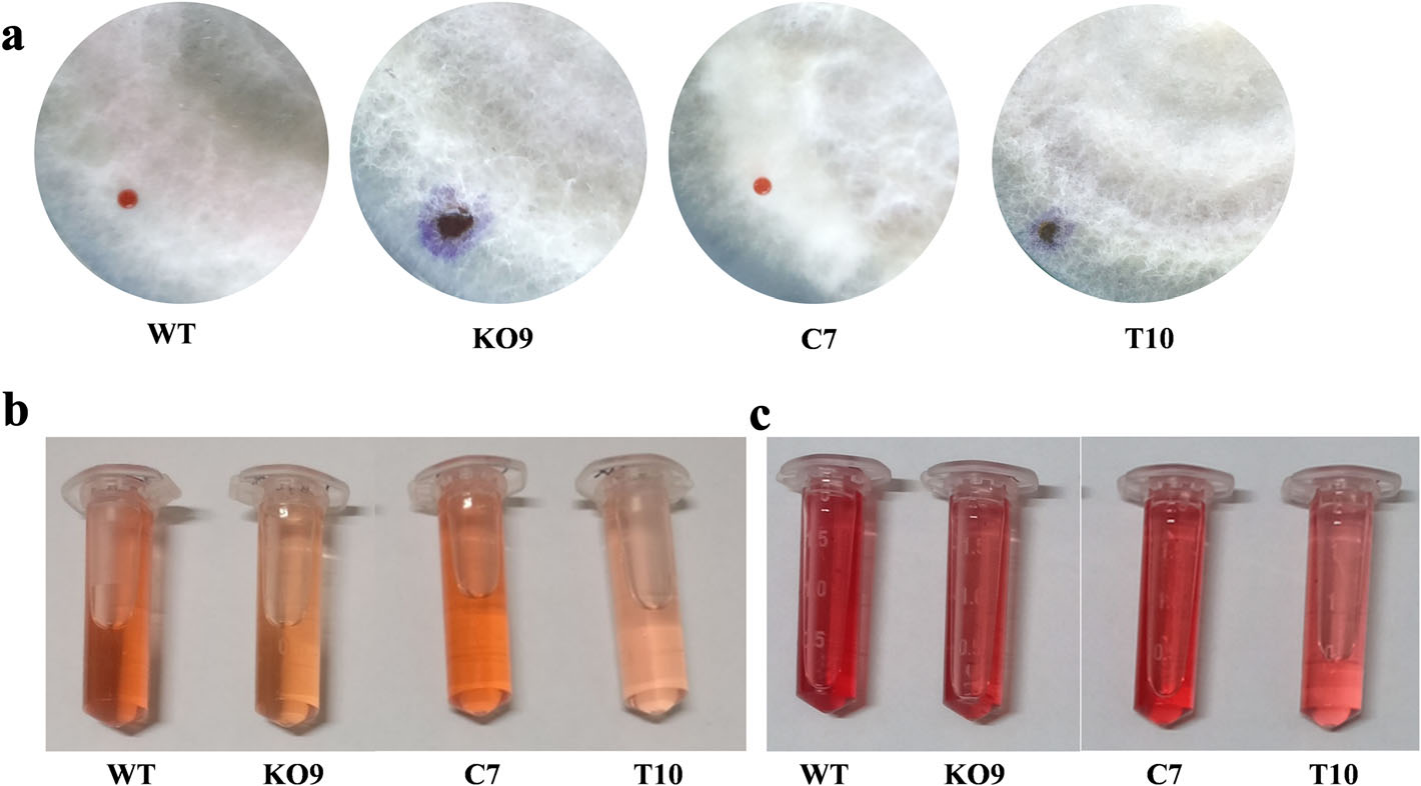
Qualitative analysis of colony hydrophobicity and pigmentation. The deletion mutant K09 and truncated mutant T10 showed loss of surface hydrophobicity as the dye percolated into the colony within 5 min whereas in wild-type and complementation mutant it was retained as droplet on the surface (**a**). Reduced pigmentation was observed in K09 and T10 mutants extracted in both ethyl acetate (**b**) and chloroform (**c**)

To assess the colony hydrophobicity, a drop of water-based dye was pipetted onto the mycelium of full-grown plates of the wild-type strain and the three mutants (K09, C7, and T10). These were observed for percolation of the dye after 5 min. The water-based dye did not percolate into the colonies of wild-type and complementation mutant (C7); however, it completely permeated into the deletion mutant K09 and truncated mutant T10 indicating that these two mutants have lost their hydrophobicity which is yet another characteristic of the aerial mycelia (Fig. 7a).

To further investigate the role of *FocSge1* in pigmentation, the pigments were extracted in two organic solvents; namely, ethyl acetate and chloroform. The amount of pigments obtained in both the solvents derived from the truncated mutant was least, followed by the deletion mutant K09. As visualized by the growth on PDA the amount of pigment in the wild-type strain was highest and was reduced with the deletion of *FocSge1* gene; but was completely restored in the complementation mutant C7 (Fig. 7b, c). These results clearly suggest that *FocSge1* is involved in mycelial pigmentation. Taken together these results reveal a crucial role for *FocSge1* in mycelial development, conidiation, colony hydrophobicity and pigmentation.

### *FocSge1* is essential for virulence of *F*. *oxysporum* f. sp. *cubense*

**Fig. 8:**
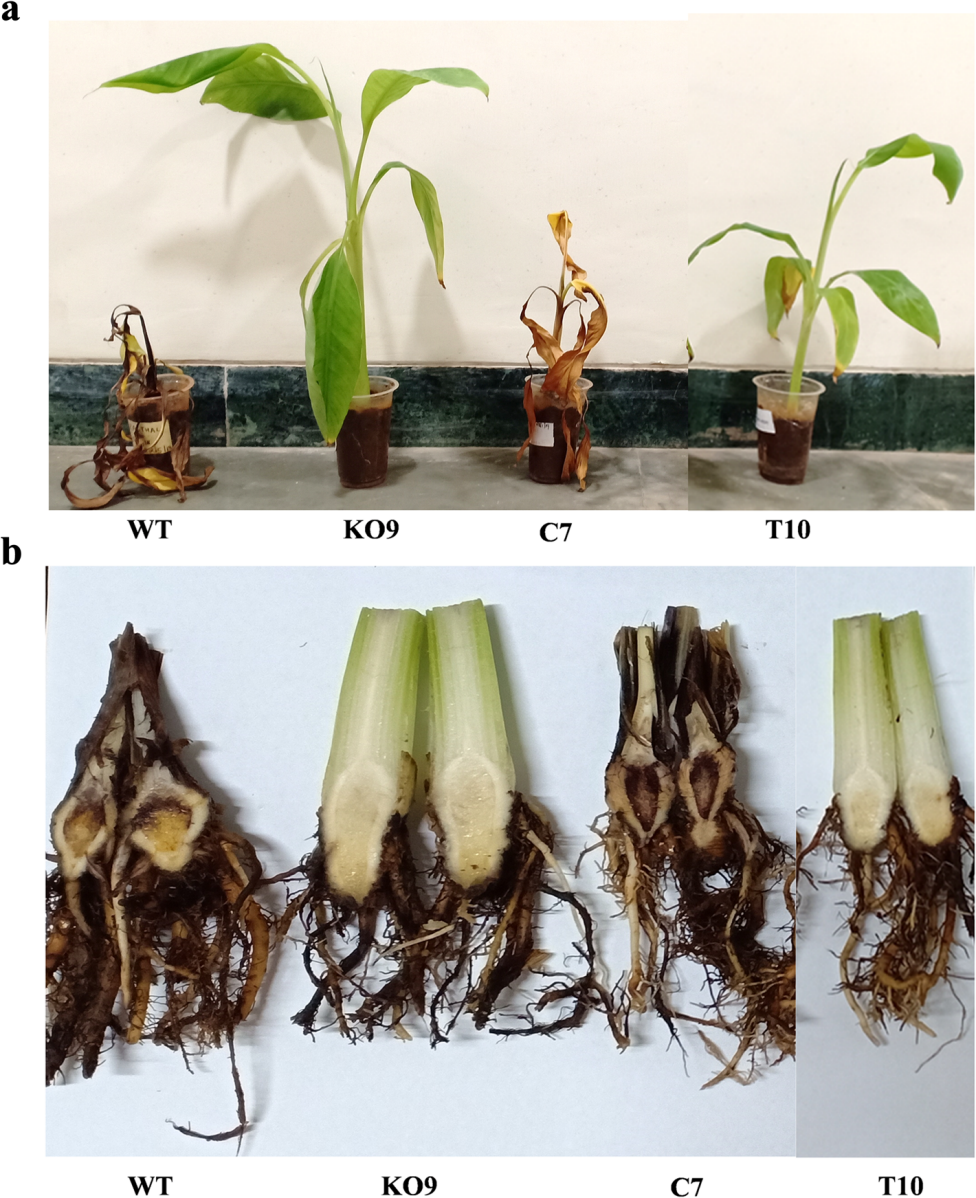
Ex vivo bioassay using susceptible banana plants. Banana plants inoculated with the wild-type and mutant strains were observed for Fusarium wilt symptoms 6 weeks post inoculation wherein the plants inoculated with wild-type and C7 mutant showed severe wilting. The plants inoculated with deletion mutant K09 and truncated mutant T10 showed no external symptoms (**a**). Longitudinal sections of the corm tissue displayed internal symptoms such as discoloration and necrosis. Severe infection was seen in plants inoculated with wild-type and C7 complementation mutant whereas plants inoculated with deletion and truncated mutants showed clean corm tissue (**b**)

To further investigate the importance of *FocSge1* gene in pathogenicity, the Foc race 1 susceptible banana plants cv. Rasthali were inoculated with four different Foc mass cultures prepared for each mutant and wild-type. This infection-based bioassay was performed in triplicates with a minimum of three plants per experiment per strain. Fusarium wilt symptoms such as yellowing of the older leaves and cracking of pseudostem was evident from third week onwards in the wild-type and C7 mutant inoculated banana plants. However, no such symptoms were observed in plants inoculated with the deletion mutant K09 and truncated mutant T10. After 6 weeks, the plants were photographed and the experiment terminated (Fig. 8a). The banana plants inoculated with wild-type strain showed severe wilt symptoms and completely wilted within the stipulated period; whereas, the plants inoculated with the deletion mutant K09 did not show any external symptoms. However, the lost virulence was restored on genetic complementation as symptoms were comparable in the banana plants infected individually with the wild-type and C7 mutant strain. The banana plants inoculated with the truncated mutant T10 also did not show any significant symptoms typical of those seen in the wild-type plant after 6 weeks. The corm tissue of all the inoculated banana plants were cut longitudinally to examine the internal symptoms. Banana plants inoculated with wild-type and C7 mutant showed maximum infestation whereas no visible internal symptoms were observed in the deletion mutant K09 and truncated mutant T10 (Fig. 8b). Taken together, *FocSge1* is required for pathogenicity on banana plants and the C-terminal domain of FocSge1 is essential for this function.

### Sensitivity of the *FocSge1* deletion mutant to osmotic stress, oxidative stress and cell wall-damaging agents

**Fig. 9:**
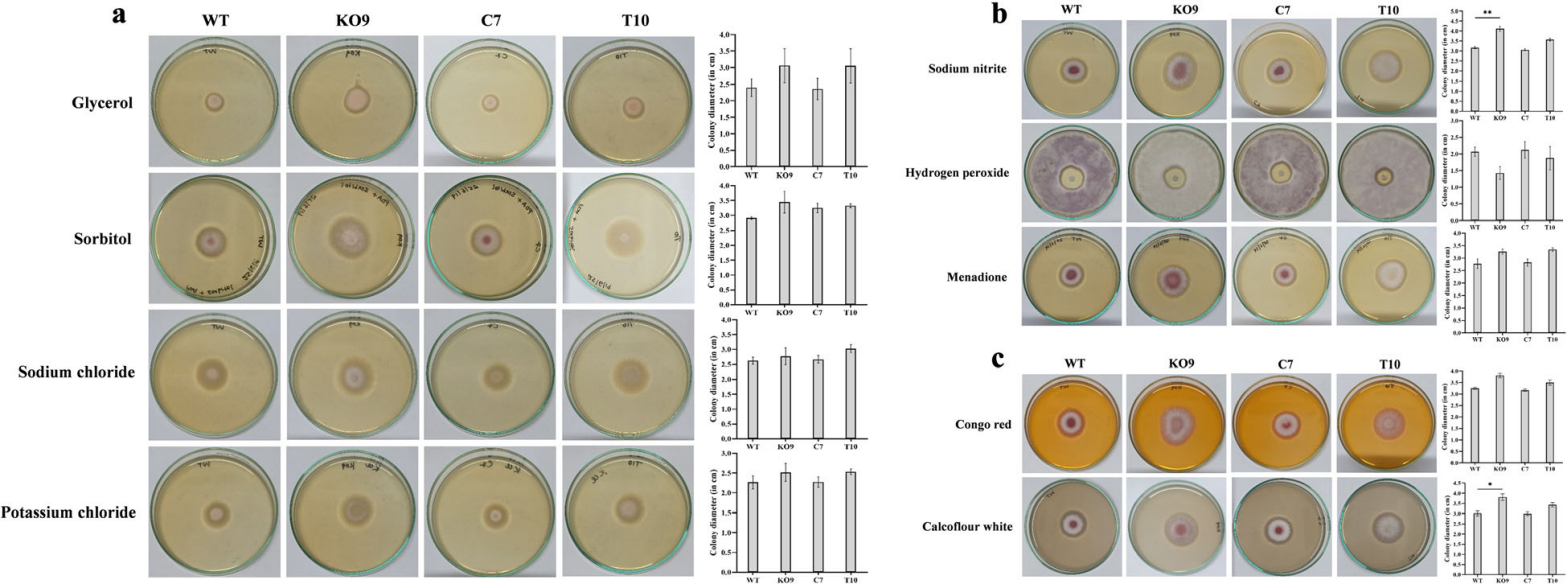
Effect of osmotic, oxidative and cell wall-stress causing agents. No significant difference in the radial growth was observed in presence of glycerol, sorbitol, sodium chloride and potassium chloride between wild-type, deletion mutant K09, complementation mutant C7 and truncated mutant T10 (**a**). The deletion mutant demonstrated improved oxidative stress tolerance in presence of sodium nitrite (**b**). In presence of cell wall stress agents such as Congo red and calcofluor white the deletion mutant showed bigger radial growth as compared to its counterparts in presence of calcofluor white (**c**). Dunnett’s multiple comparisons test was performed to determine the significant difference between the wild-type and mutant strains (** *P* < 0.01, * *P* < 0.05)

As deletion of *FocSge1* gene was known to reduce virulence, its role towards osmotic, oxidative and cell wall stress causing agents was subsequently assessed. There was no statistically significant difference in the radial growth between the wild-type and mutants (at *P* value ≤0.05) in the medium containing osmotic stress agents. However, at *P* value ≤0.1, there was significant difference between the wild-type and K09 mutant. In the sorbitol-based medium, pigmentation at the colony center was clearly seen in wild-type and C7 mutant, but, was completely absent in other 2 mutants. Moreover, a compact growth was evident in wild-type and C7; whereas, more of dispersed mycelial growth was seen in K09 and T10. (Fig. 9a).

There was statistically significant difference between wild-type and K09 mutant in the medium containing sodium nitrite. The radial growth of K09 in minimal medium supplemented with sodium nitrite and menadione was more than the wild-type. However, the sensitivity reverted back to the wild-type in the complementation mutant C7. However, at *P* value ≤0.1 there was significant difference in the zone of inhibition in medium containing hydrogen peroxide indicating that the deletion mutant is more tolerant to oxidative stress. Additionally, the wild-type and complementation mutant C7 developed pigmentation during incubation. No pigmentation was seen in deletion mutant K09, but slight pigmentation was evident in the truncated mutant T10 with minimum zone of inhibition (Fig. 9b).

To check for the sensitivity of the mutants to cell wall-damaging agents, the deletion mutant K09 and truncated mutant T10 showed resistance and more radial growth in medium supplemented with calcofluor white as compared to the wild-type strain. The genetic complementation mutant C7 showed a phenotype similar to the wild-type (Fig. 9c).

### Fusaric acid analysis

**Fig. 10:**
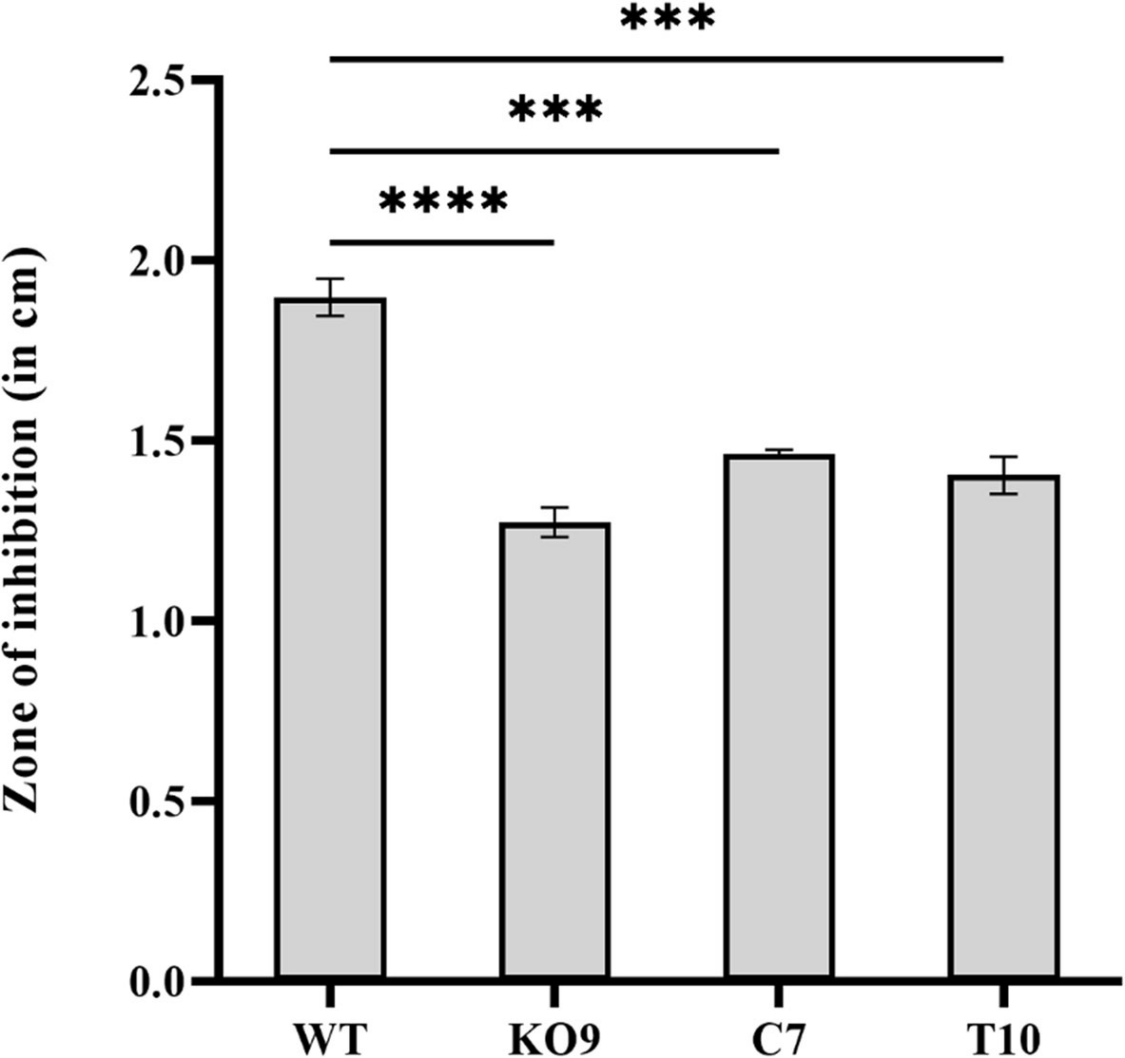
Qualitative analysis of fusaric acid produced by the strains in Czapek Dox broth. The fusaric acid extracted from the mycelia of wild-type and mutants were tested for their activity against *Bacillus subtilis* and the zone of inhibition recorded. The zone of inhibition corresponds to the amount of fusaric acid produced. The deletion mutant showed relatively smaller zone of inhibition as compared to the wild-type strain. Dunnett’s multiple comparisons test was performed to determine the significant difference between the zone of inhibition of the wild-type and mutant strains (**** *P* < 0.0001, *** *P* < 0.001)

Fusaric acid produced by the wild-type and mutant strains in Czapek Dox broth was extracted using ethyl acetate and half the fraction evaporated and dissolved in methanol. This methanol fraction when tested for *Bacillus subtilis* sensitivity, no zone of inhibition was observed. When the remaining half was extracted using acidified water and diethyl ether and tested for *Bacillus subtilis* sensitivity, a clear zone of inhibition was observed. The diameter of the zone of inhibition was highest for fusaric acid obtained from wild-type strain, followed by complementation C7 and truncated T10 mutants. A smaller zone of inhibition was observed when fusaric acid produced from deletion strain was used, indicating less production of fusaric acid (Fig. 10). These results thus indicate that *FocSge1* gene regulates the biosynthesis of fusaric acid to some extent.

## Discussion

Fusarium wilt disease of banana is a notoriety in the agricultural history known to mankind and is still threatening banana production worldwide. The rampant spread of Foc both race 1 and Tropical race 4 all throughout the major banana growing regions is creating a havoc [1]. Almost all the varieties grown for domestic consumption and export are susceptible to these pathogenic strains. Moreover, the physical and chemical methods of management are insufficient. The infected banana fields are rendered useless for fresh plantations as the dormant chlamydospores survive in the soil for decades. Besides, Foc also survives in the asymptomatic alternate hosts and does not show any Fusarium wilt symptoms [11]. Foc shifts its lifestyle from saprophytic to parasitic mode after coming in contact with the host roots which is associated with major alteration in expression and metabolite profile. This change is brought about by a few master regulators that perceive the signals and control the expression of downstream repertoire of genes required for pathogenicity [5]. *FocSge1* is one such master regulator that triggers the expression of effector genes upon colonization. Understanding the molecular basis of Fusarium wilt disease remains the major goal in plant-pathogen interaction research thereby paving way for the development of novel strategies in disease management. In the present study, we examined the roles of *FocSge1* gene in Foc race 1 that is known to infect many cultivars of banana. Disruption of *FocSge1* gene resulted in several phenotypic variations as compared to the wild-type strain. Moreover, the deletion mutant displayed loss of virulence in susceptible banana variety. We also generated complementation mutants C7 and T10 in the course of the experiments; where C7 being a complete rescue. The T10 mutant truncated at its C-terminal domain was created to demonstrate its critical role. To further assess the expression of *FocSge1* gene in wild-type and mutant strains, they were inoculated into banana cell suspension cultures. Since the effector genes are regulated even in the presence of plant cells, banana cells were chosen in this assay [5]. Expression of *FocSge1* was recorded in wild-type and complementation strains exposed to banana cells indicating that *FocSge1* responds to the presence of banana cells. Moreover, expression of the truncated form of *FocSge1* was also observed indicating that it is expressed but the deleted C-terminal domain is crucial for the lost phenotypic characteristics seen in wild-type and the complementation strains. Thus, our study suggests that *FocSge1* gene is critical for pathogenicity and regulation of normal growth and development.

*Sge1*, a homolog of *Wor1* is required for parasitic growth and virulence in *Fusarium oxysporum* f. sp. *lycopercisi* (Fol) that infects tomato [5]. Deletion or inactivation of SGE1 resulted in reduced pathogenicity and was correlated with the reduced levels of toxin production and effector proteins. SGE1 from Foc TR4 was shown to regulate series of proteins required for infection. A SGE1 deletion mutant of Foc TR4 demonstrated impaired conidiation and complete loss of virulence on banana plantlets [6]. However, *Sge1* deletion mutant of Fol showed no loss of colonization capability, whereas colonization was compromised in *Sge1* mutant of Foc. A homolog of *Sge1* in *Fusarium verticillioides* (SGE1) is required for pathogenicity and affected synthesis of secondary metabolites such as fumonisins and fusarins [7]. Nonetheless, SGE1 in *F. verticillioides* was not essential for conidiation as it was in Foc and Fol. None of these homologs were shown to affect vegetative growth of the fungus. This indicates that the role of Sge1 is varied in different phytopathogens and a need to elucidate its role for each pathosystem is imminent. Multiple sequence alignment of Sge1 protein sequences from different species revealed that the sequence is highly conserved signifying its importance in virulence. Also, Fo47 strain has additional proline-glutamine residues in the C-terminal domain. Fo47 is an endophyte but a non-pathogenic strain capable of inducing defense response in the host plants. Probably an addition of these proline-glutamine residues might have rendered structural deformity of this protein thereby contributing towards lost pathogenicity. This observation concurs with the loss of pathogenicity of the truncated mutant T10 created in this study.

Some virulence-related genes are induced by nitrogen starvation and also by the kind of nitrogen source provided in the medium [12]. The kind of nitrogen available *in planta* is different from that in soil and thus there is a differential regulation with respect to the nitrogen source available. In order to find whether *FocSge1* is regulated by nitrogen source, we firstly identified the presence of a GATA element in the promoter region of this gene. Nevertheless, the expression of the effector gene *SIX1*, which is downstream to *FocSge1* was observed in medium containing sucrose and potassium nitrate, thus we believe that *FocSge1* is in turn regulated by nitrate in the medium [13].

Deletion of *FocSge1* gene resulted in altered growth and development in Foc indicating that it influences these biological processes in this fungus. The wild-type strain showed more of a cottony growth and pigmentation on PDA and YPD media, whereas the deletion and truncated mutants displayed poor aerial hyphae and reduced pigmentation. Additionally, the radial growth was comparable with the wild-type strain in all media tested. Since we now know that *FocSge1* is differentially regulated by the kind of nitrogen source, we tested the growth phenotype of these mutants in minimal medium supplemented with different nitrogen sources. In all the media tested, the deletion mutant K09 and truncated mutant T10 displayed altered phenotype as compared to the wild-type strain in terms of mycelial growth, aerial hyphae and pigmentation. This indicates that *FocSge1* gene is involved in asexual growth development. Furthermore, the number of conidia in the deletion mutant were considerably less than the wild-type strain. The conidia count was restored when the deletion mutant was complemented with the full-length *FocSge1* gene but not in the truncated mutant. This clearly indicates that *FocSge1* is required for maintaining the conidial count. In previous study, a similar phenotype was seen in *Fusarium oxysporum* f. sp. *lycopersici* where the *SGE1* deletion mutants produced about 6-fold less microconidia compared to the wild-type [5] and the *Fusarium oxysporum* f. sp. *cubense* TR4 produced about 4-fold less conidia compared with WT [6]. However, *SGE1* deletion did not affect conidiation in *Fusarium verticillioides*, a maize pathogen [7].

The aerial hyphae and the conidia are the ones which provide hydrophobicity to the colony, since there was poor hyphal growth and conidiation, we tested the mutants for hydrophobicity and found that the deletion and truncated mutants completely lost the hydrophobicity. The pigments produced in the wild-type strain were abundant as compared to the deletion and truncated mutants. Also, this lost phenotype was regained in the complementation mutant strain.

To investigate the role of *FocSge1* in pathogenesis, 2 month-old hardened susceptible banana plants cv. Rasthali were independently inoculated with the four mass cultures (WT, K09, C7 and T10). The banana plants inoculated with the wild-type strain showed typical Fusarium wilt symptoms whereas the deletion mutant K09 could not infect the banana plants and therefore the plants did not show any external or internal symptoms. However, the virulence was restored by genetic complementation indicating that *FocSge1* is required for full virulence. Moreover, the truncated mutant T10 also showed reduced virulence. The series of altered phenotype seen in the deletion mutant including hyphal growth, hydrophobicity, poor conidial count and cell wall integrity may have together contributed to the reduced virulence in the deletion and truncated mutant on the banana plants. We tested the expression of the target gene *FocSix1* in the inoculated plants using quantitative RT-PCR where there was expression observed in the wild-type and C7 mutant whereas no expression was seen in the K09 and T10 mutant (data not shown).

Rigidity of the fungal cell wall, osmotic and oxidative stress tolerance are important characters required for maintaining cellular integrity, survival and penetration in the host cells [14–16]. Moreover, the phytopathogenic fungi also need to encounter oxidative stress generated while infecting the plant cells [17, 18]. The *FocSge1* deletion mutant K09 generated in this study showed increased tolerance to oxidative stress (sodium nitrite) as compared to the wild-type strain. Since these features are somewhat associated with the cell wall integrity, the tolerance of the mutant to cell wall-damaging agents such as Congo red and calcofluor white were tested. The radial growth in the calcofluor white medium was relatively more in the deletion and truncated mutants as compared to the wild-type strain indicating that they are capable of resisting it. Inappropriate accumulation of trehalose, glycerol and other osmolytes have been known to regulate stress responses [19–22]. Intercellular trehalose plays a critical role in free radical scavenging, whereas glycerol affirmatively responds to osmotic stress [23]. Increased tolerance of the deletion and truncated mutants to oxidative and cell-wall stress may be due to altered cell wall features and composition and thus shows that *FocSge1* is required for maintaining normal cell wall integrity. Moreover, HOG pathway is involved in stress management and accumulation of compatible solutes in the cell [24]. Based on the results obtained in our study we assume that *FocSge1* gene regulate the HOG pathway at some point and thus deletion of *FocSge1* resulted in loss of control on the HOG pathway imparting more tolerance to stress agents in deletion mutant as compared to wild-type and complementation mutant. Further the role of *FocSge1* in fusaric acid production was assessed as fusaric acid is one of the virulence factor [25]. The fusaric acid production was tested in wild-type and mutant strains, where the deletion mutant showed less production capability as compared to the wild-type and complementation mutants.

## Conclusions

In conclusion, the present work demonstrates that *FocSge1* is vital for pathogenesis in banana and is involved in hyphal growth, pigmentation, hydrophobicity, conidiation and fusaric acid production. We demonstrate that the C-terminal domain of *FocSge1* is important for its in vivo functioning as the truncated mutant behaved similar to the deletion mutant.

## Methods

### Strain and culture conditions

*Fusarium oxysporum* f. sp. *cubense* (Foc) race 1 wild-type isolate (VCG 0125) obtained from the Indian Institute of Horticulture Research (IIHR), Bangalore (India) was used in all the experiments. It was stored as a conidial suspension in 15% glycerol at − 80 °C. The wild-type strain and transformants were grown and sub-cultured on potato dextrose agar (PDA). For experiments such as conidial count, genomic DNA isolation, RNA isolation, fusaric acid extraction, Foc was grown in potato dextrose broth (PDB) at 30 °C and agitated at 160–200 rpm for 5–10 days.

### In silico analysis of *FocSge1* gene

The *FocSge1* (FOC1_g10010072) sequence was identified in the whole genome shotgun sequencing database (KB730034 AMGP01000000) of *Fusarium oxysporum* f. sp. *cubense* race1 (scaffold56, whole genome shotgun sequence) available with the NCBI database. The *FocSge1* amino acid sequence was aligned with its orthologs using the online Clustal™ Omega tool. Two kilobases upstream sequence (the promoter region) of *FocSge1* gene was screened for the presence of GATA sequence which is known to be responsive to different nitrogen sources in the medium.

### Generation of *FocSge1* deletion mutants

The *FocSge1* deletion vector was generated by inserting the upstream and downstream flanking sequences of *FocSge1* onto the two sides of the hygromycin resistance gene cassette. The *FocSge1* upstream sequence (2047 bp) along with 162 bp of the coding region was amplified using *FocSge1* UPS Fw/*FocSge1* UPS Rv primer set and inserted into the *Kpn*I and *Xho*I restriction sites of the pCSN44 vector backbone upstream to the hygromycin cassette. Subsequently, the downstream flanking sequence of 1998 bp of *FocSge1* along with 124 bp of coding sequence was amplified using *FocSge1* DWS Fw/*FocSge1* DWS Rv primer pair and cloned downstream of the hygromycin cassette between *Not*I and *Xma*I sites to form the pCSN44-∆*FocSge1* deletion vector. Both upstream and downstream DNA fragment of *FocSge1* gene were sequenced. This pCSN44-∆*FocSge1* deletion vector was transformed into Foc spheroplasts.

The spheroplasts were prepared by growing the Foc culture on potato dextrose agar at 30 °C for 8–12 days. Conidia were harvested using Tween 80 solution (0.005% v/v) and separated from the mycelia by filtering through the absorbent cotton bed. Approximately, 10^8^ conidia/mL were inoculated into potato dextrose broth and incubated at 30 °C and agitated at 200 rpm for 22–23 h. The following day, mycelia was separated using four layers of cheese-cloth and washed several times with chilled distilled water. Further the mycelia was washed with chilled osmotic medium (0.27 M CaCl_2_ and 0.6 M NaCl). The washed mycelia was then transferred to a flask containing osmotic medium, lysing enzyme [900 mg/mL] (Novozyme, USA) and BSA fraction V [60 mg/mL] (MP Biomedicals, USA) and mixed to form a homogenous slurry. This mixture was then incubated in water bath at 37 °C for 2–3 h. The reaction mixture was swirled vigorously to release protoplasts from the mycelial debris. This reaction mix was then filtered through Mira cloth (Merck, USA) to separate the spheroplasts from the mycelial debris. The spheroplasts were then pelleted by centrifuging at 1650 *g* in a swinging bucket rotor for 10 min. The pellet was washed with 1X STC solution (1.2 M D-sorbitol, 50 mM CaCl_2_ and 35 mM NaCl, 10 mM Tris). The supernatant was discarded and pellet was resuspended in 1X STC. Five microgram of the linearized plasmid DNA (using *Pci*I) along with equal volume of 2X STC, 50 μl 50% w/v PEG (t) (2X STC: 50% w/v PEG 8000::1:1) and W7 hydrochloride (5 μg/mL) [26] was added to the spheroplasts and incubated on ice for 30 min. After incubation 1 mL of PEG(t) was added to the mixture and further incubated at room temperature for 30 min. This mixture was then spread onto PDA medium containing sucrose (1.329 M) and incubated at room temperature. After 16–18 h, the plates were overlaid with PDA medium supplemented with hygromycin (50 μg/mL) and incubated at 30 °C for 2–3 days till the appearance of transformants on the plates. The putative transformants were then grown on PDA medium supplemented with hygromycin and screened by PCR using primers *FocSge1*cds Fw and *FocSge1*cds Rv. A single confirmed transformant was selected and used for further analysis.

### Complementation of the *FocSge1* gene deletion mutant with full-length gene and truncated version

To functionally annotate *FocSge1* gene, the deletion mutant strain was complemented with the full-length *FocSge1* gene. The complementation vector was constructed by PCR amplifying the locus having 2000 bp upstream promoter region, 993 bp coding sequence and 1000 bp downstream terminator region using *FocSge1* Comp Fw/*FocSge1* Comp Rv primer pair. This 4054 bp amplified sequence was cloned into *Not*I and *Bam*HI site of pBC-Phleo vector to give pBC-Phleo-∆*FocSge1C*. The *FocSge1* gene in pBC-Phleo vector was sequenced. This vector was further linearized using *Bmt*I and transformed into Foc spheroplast as described above. The transformants were selected on the medium supplemented with phleomycin and hygromycin. The transformants were sub-cultured for seven generations, further prepared into single spore suspension and stored until further use. The putative transformants were screened for the complementation construct using PCR. Simultaneously, a truncated version (pBC-Phleo-∆*FocSge1T*) of *FocSge1* gene was created using primer pair *FocSge1*CompTRN Fw/ *FocSge1*CompTRN Rv, wherein the 70 amino acids (210 bp) C-terminal domain was deleted. The pBC-Phleo-∆*FocSge1T* vector was linearized using *Apa*I and transformed into the deletion mutant as described previously to investigate its importance.

### Quantitative reverse transcription PCR (qRT-PCR) analysis

To validate *FocSge1* expression in the wild-type and mutant strains of Foc, 10^5^ conidia were inoculated into cell suspension cultures of banana cv. Rasthali. Inoculated and un-inoculated control flasks were incubated at 25 °C, 80 rpm for 60 h. Cultures were harvested by centrifugation and total RNA was extracted as previously described in [27]. The total RNA was purified using RNeasy mini kit (Qiagen, Netherlands) and DNA was removed using on-column DNase treatment, following the manufacturers’ instructions. Total RNA (1 μg) was used to prepare cDNA with ProtoScript® First Strand cDNA Synthesis Kit (New England Biolabs MA, USA) following manufactures’ protocol. Fifty nanograms of cDNA was used as template for expression analysis of *FocSge1* gene using *Sge1RT* Fw and *Sge1RT* Rv primers. qRT-PCR was performed using CFX96 Real Time System (Bio-Rad, California, United States) and SsoFast EvaGreen® Supermix (Bio-Rad, California, United States). The Foc translation elongation factor 1 α (TEF-1α) was used as an endogenous reference control for normalization and reactions were carried out in triplicates. The relative expression levels were determined using REST-MCS software [28].

### Mycelial growth on different medium

Foc wild-type and mutant culture plugs were obtained from 10 days-old PDA plate and inoculated on different media such as potato dextrose agar (PDA), Czapek Dox agar, yeast extract peptone dextrose agar (YPD) and minimal media containing different nitrogen sources (glutamine, urea, sodium nitrate, ammonium chloride, ammonium nitrate). These plates were incubated at 30 °C for 8 days. The plates were observed and documented for growth, colony morphology and pigmentation.

### Effect on pigmentation and conidiation

Conidia were harvested from a 10 days-old Foc plate and counted using hemocytometer. Further, 10^3^ conidia were inoculated in 50 mL of PDB. This was incubated at 30 °C, 160 rpm for 10 days. Mycelia was separated using four layers of cheese cloth and ground into fine powder using liquid nitrogen. This powdered mycelia was then equally divided into two 15 mL tubes containing 10 mL each of ethyl acetate and chloroform. The tubes were incubated on a rotary shaker overnight at room temperature. The crushed mycelia were separated by centrifuging at 8228 *g* for 20 min at room temperature. This experiment was repeated at least thrice each with three technical replicates.

### Effect on colony hydrophobicity

Foc wild-type and mutant plugs were obtained from 10 days-old PDA plate and inoculated on PDA medium. The plates were incubated at 30 °C for 8–10 days and used for hydrophobicity testing. Ten microliters of a water-based dye was pipetted onto the colonies and allowed to rest at room temperature for 5 min. The percolation of the dye was observed and plates were photographed.

### Pathogenicity assay

The ex vivo bioassay was carried out using 2 months-old hardened tissue culture banana plants cv. Rasthali (AAB ‘Silk’ group) (derived from the micropropagation of banana from embryogenic callus) and Foc mass culture [29, 30]. Rasthali plants were obtained from fields and authenticated by the Karnataka Horticulture Department, Govt. of India. For Foc mass culture, wild-type Foc strain and the other two mutants were grown in PDB for 10 days at 30 °C at 160 rpm. Five hundred milliliters of Foc culture was inoculated in the mixture of autoclaved sand and maize bran in the ratio of 19:1. This mixture was incubated at room temperature for 4 weeks and used for infection. The hardened banana plants were uprooted, roots injured using needle and transplanted in the mixture of soil and Foc mass culture in 1:1 ratio. These plants were incubated under controlled conditions and observed for symptoms after 6 weeks. Three plants were inoculated with each strain and the experiment was repeated thrice. After 6 weeks, the plants were uprooted and corm tissue was sectioned longitudinally to observe internal symptoms.

### Sensitivity of the *FocSge1* deletion mutant to osmotic stress, oxidative stress and cell wall-damaging agents

To test the sensitivity of the Foc wild-type strain and mutants against osmotic, oxidative and cell wall stress agents, the conidia were allowed to grow in medium containing the stressors. For osmotic stress experiments, glycerol (0.8 M), sorbitol (1.0 M), KCl (1 M) and NaCl (0.8 M) were added individually to the medium. For inducing oxidative stress, menadione (10 μg/mL) and sodium nitrite (1 mM) were added individually to the medium. For hydrogen peroxide assay, 10^3^ conidia were spread on the plate and incubated at 30 °C overnight. Following day, sterile paper discs loaded with 3% hydrogen peroxide was placed in the center of the plate and incubated further at 30 °C for 2 days. For cell wall stress, Congo red (50 μg/mL) and calcoflour white (40 μg/mL) was used and mixed directly into the medium [31, 32]. Conidia were harvested from 10 days-old Foc wild-type and mutant plates and 10^3^ conidia were inoculated in the center of each plate. Plates were incubated at 30 °C for 3 days. For all the assays, radial growth was measured; while, for hydrogen peroxide assay, the zone of inhibition was measured and the difference was represented in graphical format.

### Detection of Fusaric acid production

Conidia were harvested from a 10 days-old PDA plate and 10^3^ spores were inoculated in 500 mL Czapek Dox broth and further incubated at 30 °C at 160 rpm for 10 days. Mycelia was separated using cheese cloth and 1 g (wet weight) was crushed to a fine powder using liquid nitrogen. Fusaric acid was extracted in ethyl acetate solvent using Soxhlet [33]. The ethyl acetate fraction containing fusaric acid was then divided into two parts; one part was evaporated to dryness and reconstituted in 2 mL of methanol; whereas, the other was evaporated to dryness and reconstituted in 25 mL distilled water (pH -3.0 adjusted using 3 N HCl). Further, it was extracted with an equal volume of ethyl ether 3 times that was evaporated to dryness and the residue was finally reconstituted in 2 mL methanol. Overnight grown culture of *Bacillus subtilis* was used to test the presence of fusaric acid. The *Bacillus subtilis* culture (0.1 OD_600nm_) was swabbed on the nutrient agar plate, wells bored and 50 μL of the extract was added to each well. These plates were incubated overnight at 30 °C and observed for the zone of inhibition.

### Statistical analysis

To determine the statistical significance between the two groups for all experimental values Dunnett’s multiple comparisons test was carried out using GraphPad Prism 8.0.2. (*P* value ≤ 0.05).

## Supplementary information

**Additional file 1: Table S1.** Primers used the study.

**Additional file 2: Figure S1.** Multiple sequence alignment of protein sequences of FocSge1 with gluconate transporter inducer Gti1 from *S*. *pombe* (NP_592911), Bcreg1 from *Botrytis cinerea* (XP_024547925), Ryp1 from *Histoplasma capsulatum* (ABX74945.1) and Wor1p from *Candida albicans* SC5314 (AOW26645.1).

**Additional file 3: Figure S2.** Multiple sequence alignment of protein sequences of FocSge1 with its homologs from *Fusarium oxysporum* f. sp. *lycopersici* 4287 (XP_018248224), *Fusarium* sp. FOSC 3-a (EWY91661), *Fusarium oxysporum* f. sp. *pisi* HDV247 (EXA45922), *Fusarium oxysporum* Fo5176 (EGU84598) and *Fusarium oxysporum* Fo47 (EWZ38328).

**Additional file 4.**

## Data Availability

All data analyzed during this study are included in this article. The *FocSge1* coding sequence is available in the National Center for Biotechnology Information (NCBI) database with accession number MT797862.

## Abbreviations

Foc: *Fusarium oxysporum* f. sp. *cubense*
f. sp.: Formae speciales
TR4: Tropical race 4
Fol: *Fusarium oxysporum* f. sp. *lycopersici*
SGE1: SIX Gene Expression 1
SIX1: Secreted In xylem 1
aa: Amino acids
Kbp: Kilo base pairs
PCR: Polymerase Chain Reaction
PDA: Potato Dextrose Agar
PDB: Potato Dextrose Broth
YPD: Yeast Extract Peptone Dextrose Agar
cv: Cultivar
VCG: Vegetative Compatible Groups
qRT-PCR: Quantitative Real-Time PCR
