# Supplementary material for: FocSge1 in Fusarium oxysporum f. sp. cubense race 1 is essential for full virulence
Source: BMC Microbiol. 2020 Aug 14;20:255. doi: 10.1186/s12866-020-01936-y (PMC7427899; doi:10.1186/s12866-020-01936-y)
Supplement: Supplementary file 1 — Additional file 1: Table S1. Primers used the study. [file 12866_2020_1936_MOESM1_ESM.docx]

**Supplementary Table 1**: Primers used in this study

| Primer name | Primer sequence (5’- 3’) |
| --- | --- |
| *FocSge1* UPS Fw | CACGGTACCGTCGTTAGTTATGGGCGCTG |
| *FocSge1* UPS Rv | TATCTCGAGCACCAGCACCAACCACAG |
| *FocSge1* DWS Fw | CCACCCGGGCATGTGGTCGACGAGGGAC |
| *FocSge1* DWS Rv | ATTGCGGCCGCTGCATAAGGAGGATGAAGGGAG |
| *FocSge1*cds Fw | ATGTCTGGAACCATGCCACTC |
| *FocSge1*cds Rv | CTACCACCACGCCTGACCATA |
| *FocSge1* Comp Fw | ATTGCGGCCGCGATGCATAAGGAGGATGAAGGGAG |
| *FocSge1* Comp Rv | CACGGATCCGGTTCAGATCGCTTCCTTCAGTAA |
| *FocSge1*CompTRN Fw | ATTCTCGAGCTACTGTTGTGGCTGCTGTTGGA |
| *FocSge1*CompTRN Rv | GCCCTCGAGAACGTGCTTATATAGAGCTTC |
| *Hph*cds Fw | AAAAGCCTGAACTCACCGCG |
| *Hph*cds Rv | CTATTCCTTTGCCCTCGGACG |
| *FocSge1* RT Fw | TTCATGTTCCTCGTCGACCA |
| *FocSge1* RT Rv | TACCATGTTCCAGGCCTGTT |
