# Supplementary figures and images for: FocSge1 in Fusarium oxysporum f. sp. cubense race 1 is essential for full virulence
Source: BMC Microbiol. 2020 Aug 14;20:255. doi: 10.1186/s12866-020-01936-y (PMC7427899; doi:10.1186/s12866-020-01936-y)

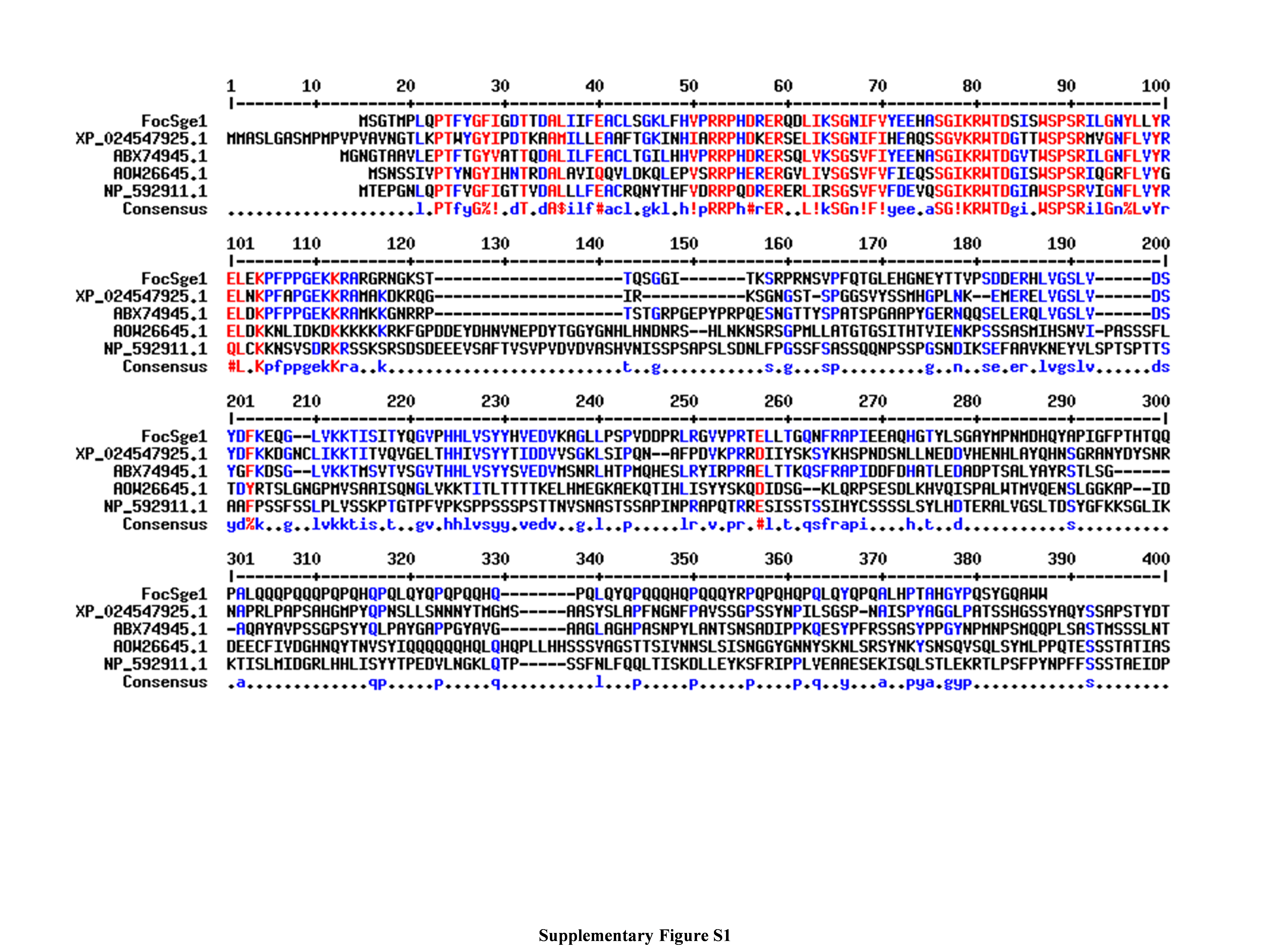

Supplement: Supplementary file 2 — Additional file 2: Figure S1. Multiple sequence alignment of protein sequences of FocSge1 with gluconate transporter inducer Gti1 from S. pombe (NP_592911), Bcreg1 from Botrytis cinerea (XP_024547925), Ryp1 from Histoplasma capsulatum (ABX74945.1) and Wor1p from Candida albicans SC5314 (AOW26645.1). [file 12866_2020_1936_MOESM2_ESM.tif]

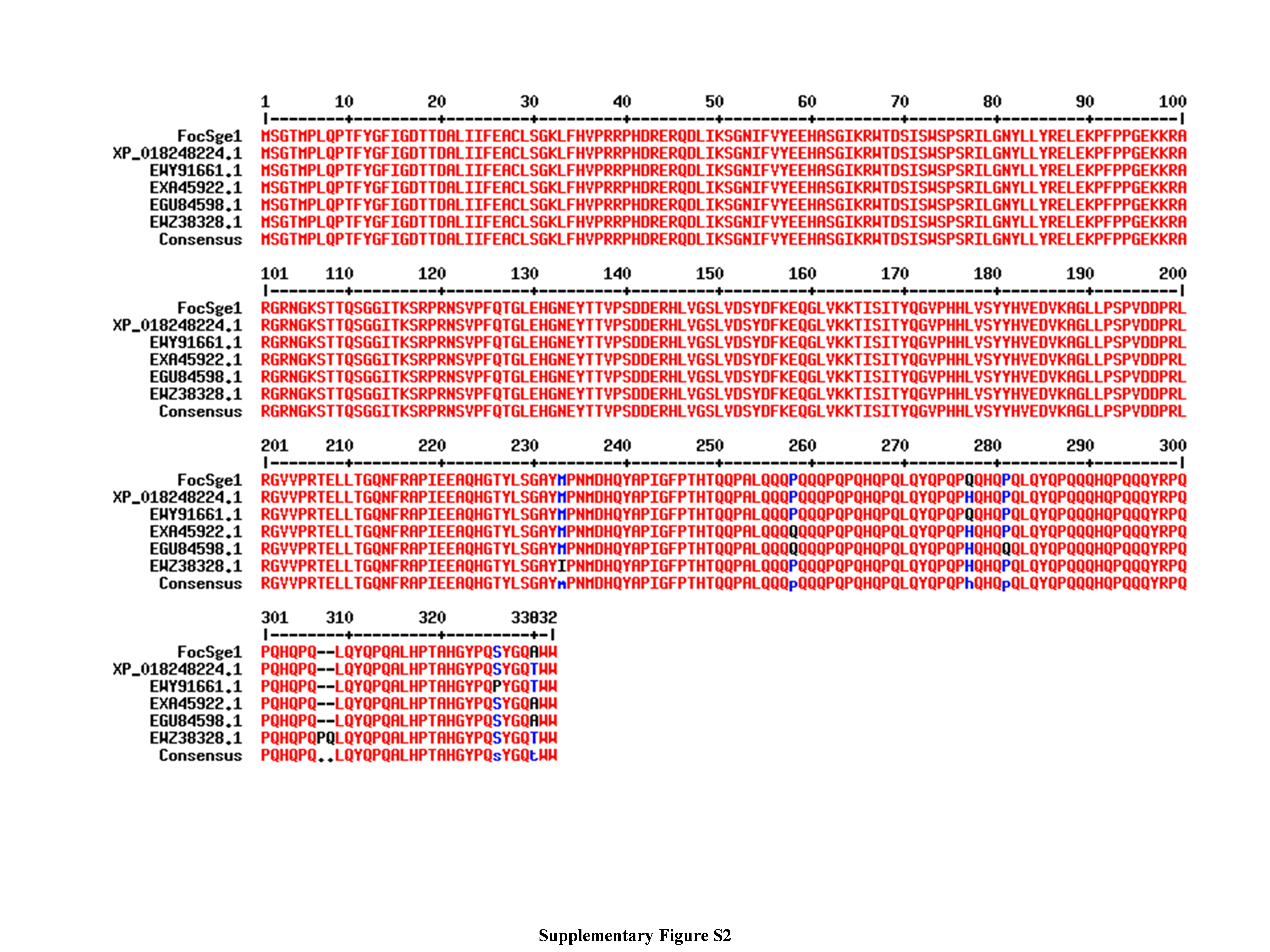

Supplement: Supplementary file 3 — Additional file 3: Figure S2. Multiple sequence alignment of protein sequences of FocSge1 with its homologs from Fusarium oxysporum f. sp. lycopersici 4287 (XP_018248224), Fusarium sp. FOSC 3-a (EWY91661), Fusarium oxysporum f. sp. pisi HDV247 (EXA45922), Fusarium oxysporum Fo5176 (EGU84598) and Fusarium oxysporum Fo47 (EWZ38328). [file 12866_2020_1936_MOESM3_ESM.tif]

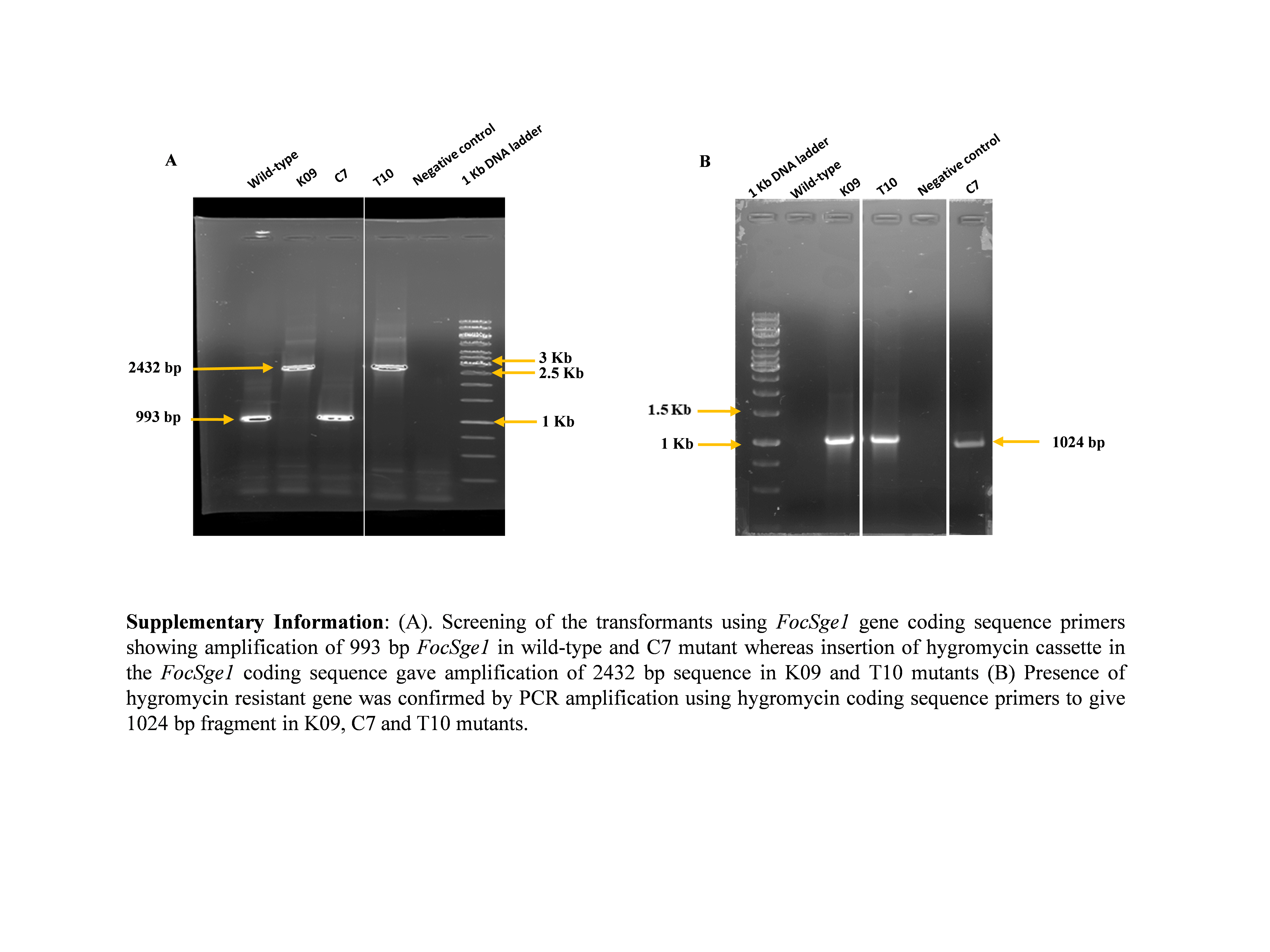

Supplement: Supplementary file 4 — Additional file 4. [file 12866_2020_1936_MOESM4_ESM.tif]
